# Fatigue-Induced Evolution of AISI 310S Steel Microstructure after Electron Beam Treatment

**DOI:** 10.3390/ma13204567

**Published:** 2020-10-14

**Authors:** Sergey Konovalov, Yurii Ivanov, Victor Gromov, Irina Panchenko

**Affiliations:** 1 Institute of Laser and Optoelectronic Intelligent Manufacturing, Wenzhou University, Wenzhou 325024, China; 2 Department of Metals Technology and Aviation Materials, Samara National Research University, Samara 443086, Russia; 3 Institute of High Current Electronics of the Siberian Branch of the RAS, Tomsk 634055, Russia; yufi55@mail.ru; 4 Department of Natural Sciences Named after Professor V.M. Finkel, Siberian State Industrial University, Novokuznetsk 654007, Russia; gromov@physics.sibsiu.ru (V.G.); i.r.i.ss@yandex.ru (I.P.)

**Keywords:** steel, fatigue, failure, electron beam treatment, structure, surface

## Abstract

Research was carried out to explore the effect of pulsed electron beam irradiation on the behavior of structure and phase state in AISI 310S steel exposed to high-cycle fatigue. A 2.2 times increase in the fatigue life of samples irradiated by electron beams was revealed. The outcomes of scanning and transmission electron microscopic studies suggest the most probable reason for the fracture of steel samples irradiated by a high-intensity electron beam to be microcraters originating on a treated surface and acting as stress risers initiating the propagation of microcracks. The irradiation with a pulsed electron beam causes extremely fast melting of the surface. As a result of the subsequent rapid crystallization, a polycrystalline structure nearly twice as small as an average grain in the untreated steel is formed. Since a surface layer crystallizes rapidly, crystallization cells ranging from 120 to 170 nm develop in the volume of grains. The fatigue testing is shown to be associated with a martensite transformation γ ⇒ ε in the surface layer. One option to intensify a fatigue life increase of the steel in focus is supposed to be the neutralization of crater-forming on a surface treated by electron beams.

## 1. Introduction

To date, there is a considerable volume of published studies exploring processes taking place when fatiguing metallic materials. It is related to a complex influence of numerous factors which cause the destruction of metals when fatigued. For instance, fatigue fracture of metals and propagation of fatigue cracks depend on stress levels and rates, surface roughness, heterogeneity of microstructure, etc. To an extent, all of these factors are relevant for the growth of fatigue cracks [1,2,3,4,5,6].

Several studies have emphasized the importance of an environmental factor in the propagation of cracks under fatigue, especially for high-duty products operated in diverse stress conditions [7,8,9]. To illustrate, Huang et al. [10] analyzed the role of a surface state in S135 steel under a variety of conditions: smooth samples, notched samples, and notched samples susceptible to H_2_S corrosion. The researchers came to a conclusion that cracks in notched and H_2_S corroded samples occurred in the early propagation phase; furthermore, a crack originates and grows due to the hydrogen-caused brittle behavior and fatigue stresses. The H_2_S corrosion produces a more significant effect on the fatigue life [10]. The fatigue fracture in SA508 steel is ductile up to the H_2_S induced corrosion. It turns to be brittle as a result of the H_2_S corrosion. A principal effect of the H_2_S corrosion is that hydrogen atoms tend to agglomerate on grain boundaries, impurities, and defects, and give rise to the origination of fatigue cracks [11].

A number of methods are known to improve a surface state responsible for microcracking [12,13,14,15,16]. The impact of an electron beam on the surface represents one of the future techniques [17]. For example, a composite nanodimensional structure was developed on a modified surface of the quenched and tempered 40CrNiMo7 steel when modifying; moreover, a martensite transformation and the dissolution and destruction of cementite were observed. As a result, the annual corrosion rate dropped from 0.12 to 0.02 mm per year, indicating, therefore, a considerable increase in the corrosion resistance [18].

The investigation on the 5CrMnMo cast steel has established a relation between the roughness behavior and microhardness of the surface in case a beam current is changed. The wear resistance of a modified layer is decidedly better than that in the initial state. In addition, a composite microstructure of needle-shaped and lath types of martensite formed owing to a martensite transformation in the modified layer [19].

The electron beam treatment of a Co-Cr base resulted in the reorientation of TiN microvolumes from (111) to (200), and, as a consequence, a three-dimensional growth mode turned into an LBL one. The process of the electron beam treatment exerts no essential effect on the thickness of coatings and deposition velocity, but the surface becomes rougher. Furthermore, an outcome of the electron beam treatment is the decreasing hardness of deposited coatings from 10 to 5 GPa [20].

The irradiation with a pulsed electron beam was used as a method to process the surface of AZ31B magnesium alloy with the purpose of improving its corrosion and wear resistance. As a result of selective evaporation of Mg induced by electron beam treatment (energy density—5 J/cm^2^, 40 cycles), a re-consolidated and Al-enriched layer formed with a nano-grained structure containing Mg_3.1_Al_0.9_ metastable phase. Such a re-consolidated layer forming due to the irradiation made it possible to reduce the corrosion rate of AZ31B magnesium alloy in 3.5% NaCl solution [21].

Principal transformations in the surface of a TiC–(Ni–Cr) metal–ceramic alloy irradiated with an electron beam are related to the development of a gradient structure, which improves wear resistance of the surface, decreases the friction coefficient, and increases the bending strength of a sample if being irradiated side of the surface is stressed [22]. Such effects, e.g., gradient layers with outstanding mechanical properties, were reported when studying a combined modification method of a tool steel surface, which involved electron beam treatment, plasma nitriding, and subsequent electron beam treatment [23].

The electron beam treatment has an essentially positive effect on the surface roughness of TiN/TiO_2_ coatings deposited via reactive magnetron sputtering on Ti5Al4V bases. It is reported that there is more symmetric distribution of heights in comparison with untreated bases [24].

Another important fact is that the electron beam treatment furthers the formation of more refined grains due to the ultrafast cooling of a melt layer; as a result, wear resistance of tools and their service life are better. However, this technique suffers from certain limitations because of residual tensile stresses in the surface layer, which, in turn, might lead to premature failures [25]. On the contrary, the electron beam treatment of Al-19.4Si titanium alloy and VT1-0 titanium by a high-intensity electron beam has resulted in a manifold increase in the fatigue life of a material [26,27].

To summarize, the electron beam treatment is a promising method for considerably improving the surface of metallic materials, which is important for processing metals and alloys exposed to fatiguing. Therefore, this study aims to explore the structure and phase composition of austenitic AISI 310S steel irradiated by an intensive pulsed electron beam and find the fracture mechanism when fatigued.

## 2. Materials and Methods

For the research, AISI 310S austenitic steel was used, and its chemical composition is given in Table 1. The steel was pretreated thermally via 2 h annealing at a temperature of 1150 °C and cooling in air.

**Figure 1:**
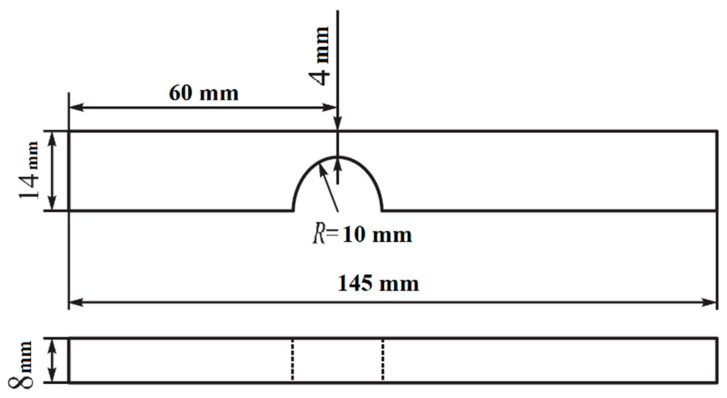
Geometry of the cycle fatigue test samples: side and top views.

Forms and sizes of samples used in fatigue testing were similar to those in studies [26,28] and are shown in Figure 1. To be precise, they were prepared as a parallelepiped with dimensions of 8 × 14 × 145 mm. The imitation of a crack was carried out by a semicircle notch with a 10 mm radius. The prior research has established that sizes of samples make no difference for the fatigue life [29].

Before carrying out fatigue tests, samples were irradiated with an intensive pulsed electron beam, using the “SOLO” laboratory unit (Institute of High Current Electronics Siberian Branch of the Russian Academy of Sciences). The operation parameters of this unit are thoroughly described in papers [17,30,31]. Parameters of our experiments are as follows: energy of electrons *eV* = 18 keV; density of the electron beam *E_S_* (J/cm^2^) = 20; pulse time τ (µs) = 50; number of pulses *n* = 3; pulse repetition frequency *f* = 0.3 s^−1^; and pressure of the residual gas (argon) in the processing chamber of the unit ~0.02 Pa.

A special assembly intended for the asymmetric cantilevered bending was used when fatigue testing (similar to that described by researchers in [32,33]). Samples were exposed to the cycling with a frequency of 20 Hz at a stress ratio of R = 0.1 (minimum vs. maximum stress in one cycle (σ = 86 MPa)). The test temperature was set 300 K. The average number of cycles required for the fracture was as high as 1.5 × 10^5^. Ten samples were examined in each irradiation mode.

The structure of steel in the as-delivered state and after fatigue tests was explored, using methods of optical and scanning microscopy and TEM electron diffraction (Philips SEM-515 and JEOL JEM-2100 F). The technique of TEM electron diffraction was applied to the investigation on layers with the irradiated surface (layer 1 further in the article) and those ~10 and ~80 µm beneath the side of the sample opposite to the stress riser and irradiated with the electron beam (layers 2 and 3, respectively).

## 3. Results and Discussion

### 3.1. Structure of the As-Delivered AISI 310S Steel

**Figure 2:**
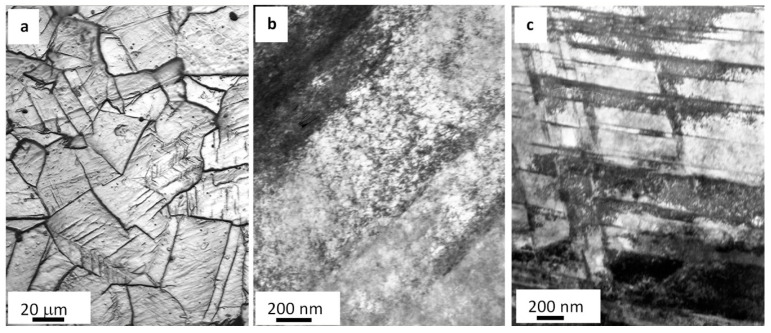
The structure of AISI 310S steel before fatiguing: (**a**) optical microscopy of an etched microsection and (**b**,**c**) TEM.

The steel of interest in the work represents a polycrystalline aggregate (Figure 2a). Grains vary from 11.4 to 88.7 µm. The mean grain size is 41.4 µm. Chaotically distributed and mesh-forming dislocations surrounded by grains were detected in the study (Figure 2b). The scalar density of dislocations (≈4 × 10^10^ cm^−2^) was determined by using the secant method [34]. A typical element in the structure of the as-delivered steel is a microtwin (Figure 2c). In most cases, microtwins attributed to one twining system were found, and, less frequently, they were members of two systems.

The steel under study is a multiphase material. Second-phase particles are detected along grain boundaries and less frequently in the volume of grains. Particles found along grain boundaries vary from 40 to 100 nm crosswise and 0.2 to 1.0 µm lengthwise. Particles in the volume of grains tend to be 0.5 µm globules.

### 3.2. Fatigue Testing Results

Our tests have revealed that the fatigue life of AISI 310S steel depends on electron beam treatment. In view of the test, specimens irradiated with the electron beam (20 J/cm^2^, 50 µs, and 3 pulses) demonstrated the fatigue life 3.3 × 10^5^ cycles (Table 2). Specimens made of as-delivered steel were destroyed after 1.5 × 10^5^ cycles, i.e., the irradiation of samples with a pulsed electron beam almost doubled the fatigue life. In light of fatigue testing outcomes, it was focused further only on data obtained when examining steel processed by pulsed electron beam (20 J/cm^2^, 50 µs, and 3 pulses).

### 3.3. Modeling the Temperature Field Developing in the Steel Surface Irradiated with a Pulsed Electron Beam

In the research context, the target represents quite an expanded plane metallic plate with similar properties; its surface area is comparable to that of a cross-section in the electron beam, whereas it is far broader than a beam-heated surface of the plate. The current density of the electron beam falling normally on the plate surface was assumed to be homogeneous. Owing to these assumptions, it was possible to use the heat-conductivity equation when searching for the temperature field within a certain range of energy density values of the electron beam. Rotshtein et al. provided a comprehensive method for calculating the time-related thermal behavior of a target irradiated with a pulsed electron beam and illustrated it with examples [35]. When examining the thermal behavior of AISI 310S steel irradiated with a pulsed electron beam, it was found out that the surface layer (5.7 µm) tends to melting (one-phase state—liquid), and the lifetime of the molten state is as long as 34 µs, provided that electron beam parameters are as follows: 20 J/cm^2^, 50 µs, and 3 pulses. The 6.2 µm layer under it is in a two-phase state (liquid + solid phases); this state exists for 81 µs. The steel is in the solid state at a more critical distance from the irradiated surface.

### 3.4. Modifying the Structure of AISI 310S Steel Surface When Irradiating with a Pulsed Electron Beam

**Figure 3:**
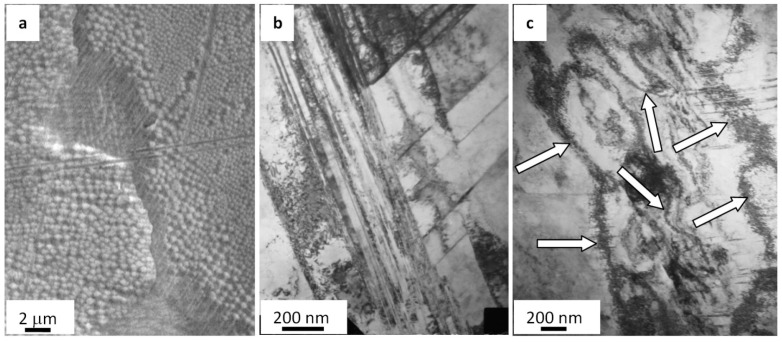
Electron microscopic image of steel surface treated with a pulsed electron beam: (**a**) SEM and (**b**,**c**) TEM.

The calculated thermal behavior of steel samples irradiated with a pulsed electron beam (20 J/cm^2^, 50 µs, and 3 pulses) indicated a high speed (6.3 × 10^7^ K/s) of the surface melting. As a result of the further rapid crystallization, a polycrystalline structure formed; its grains are 21.3 µm on average, which is approximately twice as small as an average grain in the as-delivered steel. Since the surface layer crystallizes rapidly, crystallization cells ranging 120 to 170 nm (Figure 3a) develop in the volume of grains, principally, along boundaries. In the volume of grains, there are microtwins grouping in blocks and a dislocation substructure (Figure 3b). The scalar density of dislocations is 5.7 × 10^10^ cm^−2^, which is almost 1.5 times higher than in the as-delivered steel. An important characteristic of the surface structure is the lack of second-phase fragments found when examining the as-delivered steel. Apparently, inclusions dissolve because the steel surface melts when irradiated with a pulsed electron beam.

The research into the steel structure using TEM of thin foils has pointed out extinction bend contours (Figure 3c), which indicate the crystal lattice bending–torsion in the material affected by internal stress fields. As mentioned above, a region where the liquid state of the material turns into a solid one is found at a depth of 10–11 µm. The specific characteristic of the steel in this layer is M_23_C_6_ carbide phase particles detected in the volume and on boundaries of grains. In the volume of grains, there are spherical 10–12 nm particles. They form extended micrometer long and up to 100 nm deep layers on grain boundaries.

Therefore, the irradiation of AISI 310S high-chromium austenitic steel with an intensive pulsed electron beam (18 keV, 20 J/cm^2^, 50 µs, 3 pulses, and 0.3 s^−1^, residual gas (argon) pressure in the processing chamber of the unit ~0.02 Pa) makes a thin surface layer (≈10 µm) melt rapidly. The melting of the surface layer initiates the dissolution of second-phase impurities. The investigation revealed carbide phase particles in the volume of grains and on their boundaries in the intermediate layer (liquid phase—solid-phase state).

### 3.5. Structure and Phase Analysis of Fatigue-Induced Fracture in AISI 310S Steel Untreated with an Electron Beam

**Figure 4:**
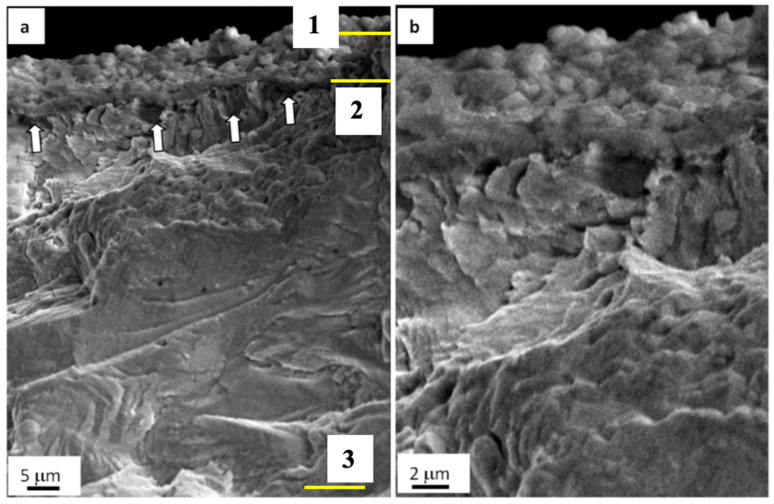
Fractography of the fatigue-fractured surface in AISI 310S steel. Arrows indicate micropores. Numbers show the position of steel objects (films) under TEM study. (**a**) general view; (**b**) enlarged photo.

The fatigue testing of the as-delivered steel (unexposed to the electron beam treatment) caused the fracture of samples after ~1.5 × 10^5^ loading cycles. Figure 3 presents the SEM data on fractured steel surfaces. As seen in the data, structure transformations are most significant in the interval 10–12 µm deep surface. Fine sizes of disintegration crystallites are typical for the forming layer (from 0.5 to 1.0 µm), which is separated from the main volume, and numerous micropores are detected along this boundary (shown by the arrow in Figure 4a).

**Figure 5:**
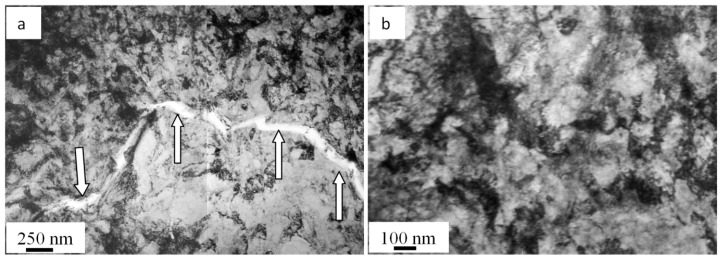
Microcracks forming along boundaries of grains containing second-phase impurities (**a**) fragmented substructure (**b**) in fractured AISI 310S steel when fatigued.

The structure-phase state of the surface was investigated by using the methods of TEM. Objects of interest (foils) were placed parallel to the front surface of a sample, i.e., opposite to the side with a stress riser, ~1, ~10, and ~80 µm away from the front surface and maximally close to the fractured surface. TEM-investigated objects are numbered in Figure 5. The research on the phase composition and defect substructure of foils taken from layer 1 (Figure 4a) has disclosed certain transformations in the steel state.

To begin with, volumes of steel with a nanodimensional structure are disclosed. In most cases, these zones are found in junction points of two microtwin systems. Crystallites in these zones vary from 25 to 80 nm. The second important outcome is that there are extended microcracks along boundaries of grains containing thin second-phase layers (Figure 5a).

Moreover, a band substructure developed in the volume of grains without microtwins when fatigue testing (Figure 5b). The bands are fragmented; fragments are in the range from 50 to 100 nm. Reflexes in the electron diffraction microphotograph taken on such a structure are indistinct, which highlights the azimuthal disorientation in the bend structure and fragments. The azimuthal component in the full angle of the substructure disorientation is estimated to be around 16 degrees.

**Figure 6:**
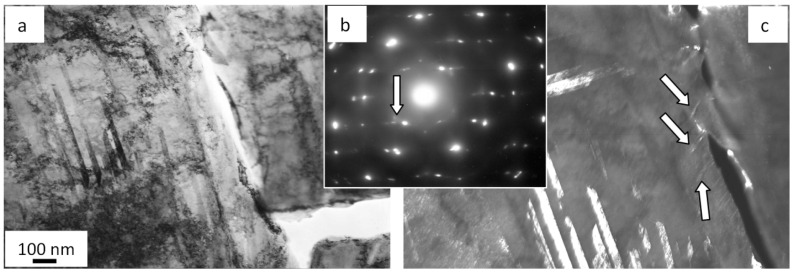
Crystals of ε-martensite forming close to grain boundaries on the surface of the fatigue-fractured AISI 310S steel; (**a**) bright field; (**b**) electron diffraction micropattern, with the arrow indicating reflexes where a dark field is obtained; (**c**) dark field recorded in the closely located reflexes [111]γ-Fe + [002]γ-Fe + [101]ε-Fe. The arrows (**c**) point at ε-martensite crystals.

In addition, the martensite γ ⇒ ε transformation is observed in the surface layer in the process of fatigue tests. Crystals of ε-martensite are detected near grain boundaries (Figure 6). Interestingly, the formation of ε-martensite is attributed to cracking. In most cases, microcracks are along grain boundaries where ε-martensite lamellae are found. Thus, it is assumed the martensite transformation is initiated by elastic stresses developing close to grain boundaries.

Finally, the fatigue-induced fracture of steel is the reason for the intensive microtwinning and, as a consequence, the complex bending–torsion of the steel crystal lattice. Electron microscopic image data on such grains demonstrate numerous extinction bend contours of a variety of shapes and sizes, which are clearly indicative for the development of inner stress fields in the steel.

Grains with a nanocrystalline structure similar to that detected in the surface are observed in an approximately 10 µm deep layer (Figure 4a, 2). However, the quantity of these grains is rather low. Therefore, a nanocrystalline structure forms in the fractured zone of AISI 310S steel in the layer to ~10 µm deep, as seen in SEM-based analysis data on the steel fractured surface (Figure 4). The principal substructure developing in the ~10 µm deep layer represents microtwins of one or two twinning systems. Identically, microtwinning is suggested to be the key deformation mechanism of steel in the ~80 µm deep layer (Figure 4a, 3). In most cases, only one twinning system is identified in grains. Twins are arranged as lengthy packages and expand almost over the entire volume of grains.

As is known, a fragmented substructure is an origin zone of the ductile fracture in a material. We consider this process in view of the following evolution scenario. While a fragmented structure is exposed to deformation, local zones unable for the further evolution (a so-called critical structure) develop in it [36]. The research on this phenomenon has pointed out that microcracks open along certain boundaries and may stop near particular joints [36,37]. As a rule, these boundaries represent boundaries with a significant plastic displacement of neighboring regions in a crystal (above 15–20 degrees), i.e., the rotational instability as detaching disclinations develop in such zones of a sample under deformation. In view of the research outcomes on AISI 310S steel and the information presented above, we conclude that several regions with a critical structure unable for the further evolution form in the approximately 10 µm deep surface layer of the fatigued steel. The development of this structure may initiate the origin and propagation of microcracks and fracture of the whole sample, as a consequence.

Another factor furthering the fatigue fracture in the steel of interest is second-phase particles (mainly those of M_23_C_6_ carbide). The deformation incompatibility of particles and matrix is responsible for the growth of inner stress fields, which, in turn, causes cracking once a critical substructure evolves.

To summarize, the fracture of AISI 310S steel exposed to high-cycle fatigue (N = ~1.5 × 10^5^ cycles) depends on the combination of several factors: locally forming regions with a so-called critical structure unable for the further evolution, i.e., volumes of the material with a depleted plasticity (fatigue life) resource, in other words, volumes of the material with a nanocrystalline structure. The approximately 10 µm deep surface layer with a nanocrystalline structure has a distinct boundary with a volume of the material beneath it. The second important reason for the fracture in the steel under study is second-phase impurities, which represent inner stress risers promoting the propagation of microcracks.

### 3.6. Structure and Phase Analysis of Fatigue-Induced Fracture in AISI 310S Steel Treated with a Pulsed Electron Beam

**Figure 7:**
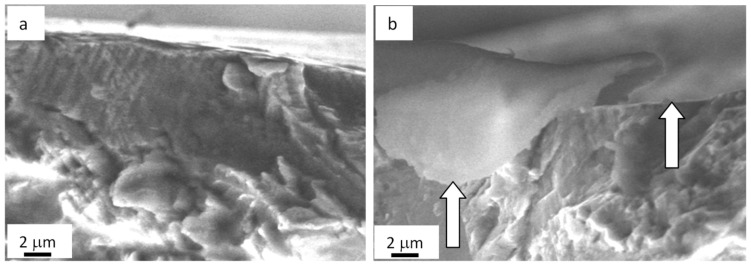
Fractography of the fatigue-fractured surface in AISI 310S steel treated with an electron beam and fractured when fatigue testing. (**a**) columnar structure. Arrows (**b**) indicate a film on the surface of steel irradiated with an electron beam.

**Figure 8:**
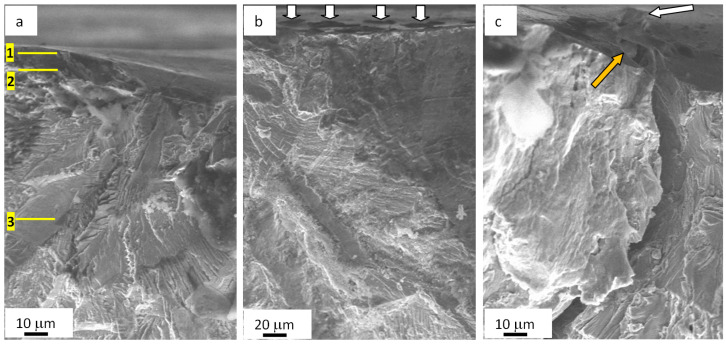
Fractography of the fatigue-fractured surface in AISI 310S steel treated with an electron beam and fractured when fatigue testing. The data in (**a**) present TEM-explored layers of a sample; arrows (**b**) highlight microcraters on the surface treated with an electron beam; a light arrow (**c**) points at a microcrater causing the formation of a mainstream macrocrack, and a color arrow indicates second-phase fragments.

The fatigue testing of steel irradiated with a pulsed electron beam has resulted in the fracture of samples after ~3.3 × 10^5^ cycles; that is twice as long as the fatigue life of steel untreated with an electron beam. The SEM-based research on the fractured surface has detected an around 10 µm deep layer with a columnar structure (Figure 7a) and a thin (≈0.5 µm) film on the surface of the specimen (Figure 7b, the film is indicated with arrows). There are microcraters on the sample surface (Figure 8b,c and microcraters are indicated with white arrows). In some cases, second-phase impurities up to 5 µm are detectable on the bottom of microcraters (Figure 8c; the impurity is shown in a yellow arrow).

To explore the phase composition and defect substructure of the fractured sample, we used electron diffraction microscopy of thin foils. Foils were prepared of lamellae found in the volume of the sample (Figure 8a). Following the data on the calculated temperature field developing in the process of electron beam treatment, we prepared foil 1 of a fragment taken from the surface layer formed in conditions of rapid crystallization; for foil 2, a fragment was used from the contact region of liquid- and solid-phase layers; and foil 3 represented the thermal impact layer. If, when bending the sample, it is assumed that the load is directed perpendicular to the surface of the sample, then the foils were made perpendicular to the direction of the load. The structure of destroyed samples was analyzed.

The investigation on the surface layer (layer 1) has revealed a polycrystalline structure. The dislocation substructure as chaotically distributed and mesh-forming dislocations is observed in the volume of grains; the cellular dislocation substructure is less frequent. The scalar density of dislocations is as high as ≈5 × 10^10^ cm^−2^. In the volume of grains, there are microtwins, which form packages of parallel lamellae.

The fatiguing leads to the bending–torsion of the steel crystal lattice. This phenomenon, seen as extinction bend contours in images of the structure, has been detected once the material structure was analyzed using the method of thin foils. The bending–torsion of the crystal lattice in the surface layer originates on grain boundaries and in microtwins. The extinction bend contour width is inversely related to the value of inner stresses. The mean crosswise size of extinction bend contours developing at boundaries of microtwins lamellae is measured to be 140 nm; that at grain boundaries is 120 nm; and at boundaries of carbide phase particles (in a range from 0.15 to 0.20 µm), it is 72 nm. Therefore, particles of carbide phase are the most significant stress risers in the steel surface under study.

**Figure 9:**
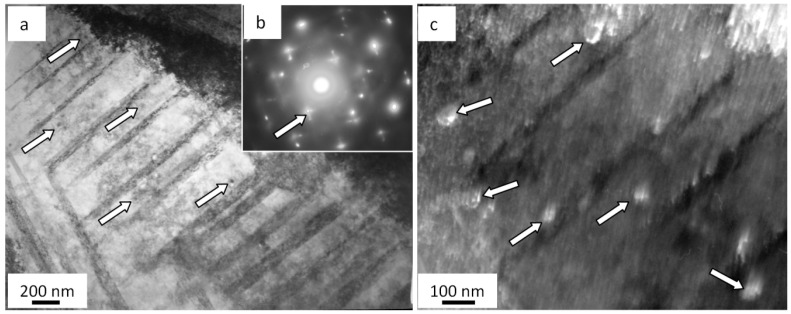
Electron microscopic image of carbide phase particles surrounded by grains in the AISI 310S steel surface processed with electron beams and fractured when fatigue testing; (**a**) bright field; (**b**) electron diffraction micropattern, a reflex where a dark field was obtained is indicated with an arrow; (**c**) a dark field obtained in similar reflexes (002)γ-Fe + (006)Cr_23_C_6_; arrows (**a**,**c**) point at carbide phase particles.

The fatiguing of samples pretreated with electron beams results in the decomposition of the solid γ-iron solution and formation of the second-phase impurities (Figure 9a). Spherical particles vary in the range of 20 to 40 nm. These particles are supposed to be chromium-based (Cr, Fe)_23_C_6_ carbide according to the indexing of the electron diffraction micropattern obtained on the volume of the material with second-phase impurities (Figure 9b).

Microtwins represent the major grain substructure in the layer at the depth of ~10 µm (layer 2, Figure 8a). Microtwins occur in grains as packages of parallel lamellae. In some cases, systems of contacting microtwins are revealed. The dislocation substructure of such grains comprises dislocation meshes with quite a high scalar density of dislocations ≈8.4 × 10^10^ cm^−2^. The percentage of the dislocation chaos substructure is insignificant.

A band dislocation structure tends to develop in grains without microtwins. Bands are arranged in slightly disoriented regions varying in the range from 150 to 300 nm. Chains of 60 to 100 nm subgrains are detected along grain boundaries. The azimuth component in the total disorientation angle of subgrains is around 12 degrees.

**Figure 10:**
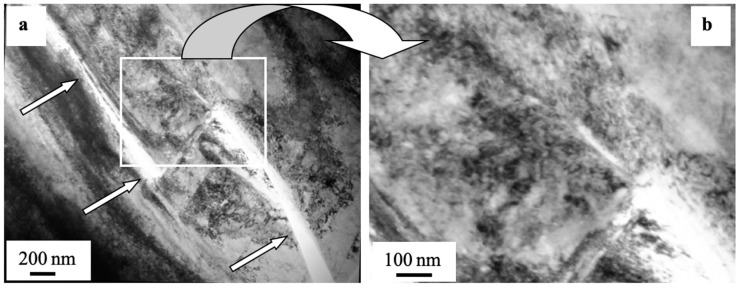
Electron microscopic image of a microcrack (indicated with the arrow (**a**)) detected in a ~10 µm deep layer in AISI 310S steel irradiated with an electron beam and fractured when fatigue testing; (**b**) shows a zoomed image of the zone presented in (**a**).

In some cases, microcracks formed in the process of preparing foils (the object of TEM-based study on the steel). Microcracks are detected mainly along grain boundaries, thus indicating the most stressed regions of the fatigued material. To illustrate this phenomenon, Figure 10 demonstrates a typical view of the structure obtained by means of electron diffraction microscopy. As a rule, lengthy second-phase layers are found on these grain boundaries. Therefore, high-level inner stress fields develop in the ~10 µm deep layer similarly to the surface, i.e., at the boundary of matrix with the carbide particle.

In a layer at a depth of approximately 80 µm (thermal impact layer), there are microtwins grouping in packages of parallel lamellae and surrounded by grains and a dislocation substructure in a form of chaotically distributed and mesh-forming dislocations. In some cases, a cellular dislocation substructure is observed along grain boundaries, and the scalar density of dislocations approximates to 6 × 10^10^ cm^−2^.

## 4. Conclusions

To sum up, the research was carried out and highlighted that the fatigue-induced fracture of untreated (not irradiated with a pulsed electron beam) AISI 310S steel is a result of locally forming zones with a so-called critical (nanodimensional) structure, unable for further evolution, i.e., with a depleted plasticity (fatigue life) resource. An approximately 10 µm deep surface layer was established to have a nanodimensional structure and a distinct boundary with a beneath-laying material. The second reason is the formation of microcracks because of inner stress risers in steel—submicron second-phase impurities.

The important outcome to emerge from this study was the operation mode of the pulsed submillisecond electron beam supply source (20 J/cm^2^, 50 µs, 0.3 s^−1^, 3 pulses, and residual gas (argon) pressure in the processing chamber, 0.02–0.03 Pa). The pretreatment of the AISI 310S steel surface in these conditions almost doubles the fatigue life of the material.

The research revealed no zones with a critical structure, i.e., regions where potential microcracks may form due to the pretreatment of samples with an electron beam (energy density of an electron beam 20 J/cm^2^ (50 µs, 0.3 s^−1^, and 3 pulses). The most probable reason for the fracture of electron beam irradiated steel samples was suggested to be the evolution of microcraters on the surface; these defects are stress concentrators promoting the formation of microcracks.

In the frame of the study, LBL electron microscopic microdiffraction investigations were conducted, which pointed out the gradient behavior of phase composition and steel defect substructure. The study revealed the substructure forming in an approximately 10 µm deep layer with a high level of inner stress fields; their maximal values were registered at the carbide-matrix boundary. In view of all the facts mentioned above, it was concluded that one of the possible measures to improve the fatigue life of steel is to neutralize the crater formation process on the electron beam treatment surface.

## Figures and Tables

**Table 1:** Chemical composition (%) of AISI 310S steel (balanced by Fe).

>C	Ni	Cr	>Si	>Mn	>P
0.2	17–20	22–25	1	0.02	0.035

**Table 2:** Results of fatigue texts of AISI 310S steel.

Without Electron Beam Irradiation		E_S_ = 20 J/cm^2^; τ = 50 µs	
No. of Sample	N, Cycles	No. of Sample	N, Cycles
0101	143,432	2001	321,654
0102	161,313	2002	315,432
0103	150,124	2003	345,981
0104	153,985	2004	341,632
0105	165,409	2005	335,134
0106	143,107	2006	339,754
0107	151,184	2007	312,464
0108	149,643	2008	335,432
0109	151,210	2009	341,247
0110	154,210	2010	330,618
Average value	152,362	Average value	331,935

## References

[1] Igwemezie V., Mehmanparast A. (2020). Waveform and frequency effects on corrosion-fatigue crack growth behaviour in modern marine steels. Int. J. Fatigue.

[2] Brayshaw W.J., Cooper A.J., Sherry A.H. (2019). Assessment of the micro-mechanical fracture processes within dissimilar metal welds. Eng. Fail. Anal..

[3] Klemenc J. (2015). Influence of fatigue–life data modelling on the estimated reliability of a structure subjected to a constant-amplitude loading. Reliab. Eng. Syst. Saf..

[4] Lesiuk G., Szata M., Rozumek D., Marciniak Z., Correia J., De Jesus A. (2018). Energy response of S355 and 41Cr4 steel during fatigue crack growth process. J. Strain Anal. Eng. Des..

[5] Panin S.V., Vlasov I.V., Sergeev V.P., Maruschak P.O., Sunder R., Ovechkin B.B. (2015). Fatigue life improvement of 12Cr1MoV steel by irradiation with Zr+ ion beam. Int. J. Fatigue.

[6] Bashkov O.V., Bryansky A.A., Belova I.V., Solovev D.B. (2019). Investigation of the stages of damage accumulation in polymer composite materials. Mater. Sci. Forum.

[7] Singh R., Singh A., Singh P.K., Mahajan D.K. (2019). Role of prior austenite grain boundaries in short fatigue crack growth in hydrogen charged RPV steel. Int. J. Press. Vessel. Pip..

[8] Kim M.-C., Park S.-G., Lee K.-H., Lee B.-S. (2015). Comparison of fracture properties in SA508 Gr.3 and Gr.4N high strength low alloy steels for advanced pressure vessel materials. Int. J. Press. Vessel. Pip..

[9] Ekaputra I.M.W., Dewa R.T., Haryadi G.D., Kim S.J. (2020). Fatigue strength analysis of S34MnV steel by accelerated staircase test. Open Eng..

[10] Huang B., Ju C., Tang J., Chen Y., Li G. (2019). Research on the fatigue properties of S135 drill pipe steel under a variety of conditions. Mater. Res. Express.

[11] Huang B., Yang J., Zhang H., Liu G., Chen Y., Li J. (2018). Influence of H_2_S corrosion on rotating bending fatigue properties of S135 drill pipe steel. Trans. Indian Inst. Met..

[12] Vdovin K.N., Dubsky G.A., Deev V.B., Egorova L.G., Nefediev A.A., Prusov E.S. (2019). Influence of a magnetic field on structure formation during the crystallization and physicomechanical properties of aluminum alloys. Russ. J. Non-Ferrous Met..

[13] Okipnyi I.B., Maruschak P.O., Zakiev V.I., Mocharskyi V.S. (2014). Fracture mechanism analysis of the heat-resistant steel 15Kh2MFA(II) after laser shock-wave processing. J. Fail. Anal. Prev..

[14] Khusainov Y.G., Ramazanov K.N. (2019). Local ion nitriding of martensitic structural steel in plasma of glow discharge with hollow cathode. Inorg. Mater. Appl. Res..

[15] Ghyngazov S.A., Vasil’ev I.P., Surzhikov A.P., Frangulyan T.S., Chernyavskii A.V. (2015). Ion processing of zirconium ceramics by high-power pulsed beams. Tech. Phys..

[16] Zhang C., Lv P., Xia H., Yang Z., Konovalov S., Chen X., Guan Q. (2019). The microstructure and properties of nanostructured Cr-Al alloying layer fabricated by high-current pulsed electron beam. Vacuum.

[17] Proskurovsky D.I., Rotshtein V.P., Ozur G.E., Ivanov Y.F., Markov A.B. (2000). Physical foundations for surface treatment of materials with low energy, high current electron beams. Surf. Coat. Technol..

[18] Wang H., Li L., Qiu S., Zhai W., Li Q., Hao S. (2020). Evolution of microstructure at the surface of 40CrNiMo7 steel treated by high-current pulsed electron beam. Coatings.

[19] Wei D., Wang X., Wang R., Cui H. (2018). Surface modification of 5CrMnMo steel with continuous scanning electron beam process. Vacuum.

[20] Valkov S., Parshorov S., Andreeva A., Bezdushnyi R., Nikolova M., Dechev D., Ivanov N., Petrov P. (2019). Influence of electron beam treatment of Co–Cr alloy on the growing mechanism, surface topography, and mechanical properties of deposited TiN/TiO_2_ coatings. Coatings.

[21] Lee W.J., Kim J., Park H.W. (2019). Improved corrosion resistance of Mg alloy AZ31B induced by selective evaporation of Mg using large pulsed electron beam irradiation. J. Mater. Sci. Technol..

[22] Yu B.-H., Ovcharenko V.E., Ivanov K.V., Mokhovikov A.A., Zhao Y.-H. (2018). Effect of surface layer structural-phase modification on tribological and strength properties of a TiC–(Ni–Cr) metal ceramic alloy. Acta Metall. Sin. Engl. Lett..

[23] Ormanova M., Petrov P., Kovacheva D. (2017). Electron beam surface treatment of tool steels. Vacuum.

[24] Petrov P., Dechev D., Ivanov N., Hikov T., Valkov S., Nikolova M., Yankov E., Parshorov S., Bezdushnyi R., Andreeva A. (2018). Study of the influence of electron beam treatment of Ti5Al4V substrate on the mechanical properties and surface topography of multilayer TiN/TiO_2_ coatings. Vacuum.

[25] College D.A., Zhu Y. (2018). Alleviating surface tensile stress in e-beam treated tool steels by cryogenic treatment. Mater. Sci. Eng. A.

[26] Ivanov Y., Alsaraeva K., Gromov V., Konovalov S., Semina O. (2015). Evolution of Al–19·4Si alloy surface structure after electron beam treatment and high cycle fatigue. Mater. Sci. Technol..

[27] Konovalov S.V., Komissarova I.A., Kosinov D.A., Ivanov Y.F., Ivanova O.V., Gromov V.E. (2017). Structure of titanium alloy, modified by electron beams and destroyed during fatigue. Lett. Mater..

[28] Ivanov Y.F., Koval N.N., Gorbunov S.V., Vorobyov S.V., Konovalov S.V., Gromov V.E. (2011). Multicyclic fatigue of stainless steel treated by a high-intensity electron beam: Surface layer structure. Russ. Phys. J..

[29] Nozaki M., Sakane M., Fujiwara M. (2020). Low cycle fatigue testing using miniature specimens. Int. J. Fatigue.

[30] Ozur G.E., Proskurovsky D.I., Rotshtein V.P., Markov A.B. (2003). Production and application of low-energy, high-current electron beams. Laser Part. Beams.

[31] Ozur G.E., Proskurovsky D.I., Karlik K.V. (2005). A wide-aperture, low-energy, and high-current electron beam source with a plasma anode based on a reflective discharge. Instrum. Exp. Tech..

[32] Gromov V., Ivanov Y., Vorobiev S., Konovalov S. (2015). Fatigue of Steels Modified by High Intensive Electron Beams.

[33] Konovalov S., Komissarova I., Ivanov Y., Gromov V., Kosinov D. (2019). Structural and phase changes under electropulse treatment of fatigue-loaded titanium alloy VT1-0. J. Mater. Res. Technol..

[34] Williams D.B., Carter C.B. (2009). Transmission Electron Microscopy.

[35] Rotshtein V., Ivanov Y., Markov A. (2006). Surface treatment of materials with low-energy, high-current electron beams. Materials Surface Processing by Directed Energy Techniques.

[36] Rybin V.V., Perevezentsev V.N., Svirina Y.V. (2017). A physical model for the initial stages of the fragmentation of polycrystals in the process of developed plastic deformation. Phys. Met. Metallogr..

[37] Zisman A.A., Rybin V.V. (2002). Disclination mode in shear microband formation in plastically deformed crystals. Solid State Phenom..

